# Reconstitution of peripheral blood T cell receptor β immune repertoire in immune checkpoint inhibitors associated myocarditis

**DOI:** 10.1186/s40959-024-00230-4

**Published:** 2024-06-11

**Authors:** Peng Yan, Yanan Liu, Mingyan Zhang, Ning Liu, Yawen Zheng, Haiqin Zhang, Hao Zhou, Meili Sun

**Affiliations:** 1 Department of Oncology, Jinan Central Hospital, Shandong First Medical University, Jinan, China; 2 Cheeloo College of Medicine, Shandong University, Jinan, China; 3 Graduate School, Shandong First Medical University, Jinan, China

**Keywords:** TCR, CDR3, Immune Repertoire, ICIs-associated myocarditis, High-throughput sequencing

## Abstract

**Purpose:**

Immune checkpoint inhibitors (ICIs)-associated myocarditis was a rare yet severe complication observed in individuals undergoing immunotherapy. This study investigated the immune status and characteristics of patients diagnosed with ICIs- associated myocarditis.

**Methods:**

A total of seven patients diagnosed with ICIs-associated myocarditis were included in the study, while five tumor patients without myocarditis were recruited as reference controls. Additionally, 30 healthy individuals were recruited as blank controls. Biochemical indices, electrocardiogram, and echocardiography measurements were obtained both prior to and following the occurrence of myocarditis. High-throughput sequencing of T cell receptor (TCR) was employed to assess the diversity and distribution characteristics of TCR CDR3 length, as well as the diversity of variable (V) and joining (J) genes of T lymphocytes in peripheral blood.

**Results:**

In the seven patients with ICIs-associated myocarditis, Troponin T (TNT) levels exhibited a significant increase following myocarditis, while other parameters such as brain natriuretic peptide (BNP), QTc interval, and left ventricular ejection fraction (LVEF) did not show any significant differences. Through sequencing, it was observed that the diversity and uniformity of CDR3 in the ICIs-associated myocarditis patients were significantly diminished. Additionally, the distribution of CDR3 nucleotides deviated from normality, and variations in the utilization of V and J gene segments.

**Conclusion:**

The reconstitution of the TCR immune repertoire may play a pivotal role in the recognition of antigens in patients with ICIs-associated myocarditis.

**Supplementary Information:**

The online version contains supplementary material available at 10.1186/s40959-024-00230-4.

## Introduction

Recently, immune checkpoint inhibitors (ICIs) have become standard therapy for many solid tumors. In the meantime, inappropriate immune activation by ICIs can lead to unique adverse effects, termed immune related adverse events (irAEs) [1]. ICI-associated myocarditis is a rare irAEs with an estimated incidence of 0.06–1% [2], but with a case fatality rate of up to 25% [3]. Myocarditis, being a potentially life-threatening complication, necessitates significant attention in clinical practice. The diagnosis of this condition involves considering various factors included a history of immune checkpoint inhibitor usage, clinical symptoms, electrocardiogram findings, troponin elevation, cardiac imaging features, and/or endomyocardial biopsy [2].

Currently, the underlying mechanisms of ICIs-associated myocarditis remain elusive. However, it is hypothesized that T cell-mediated immunity plays a significant role in its pathogenesis [4, 5]. For instance, some were thought to be a relevant cancer antigen was expressed in cardiac cells or molecular mimicry in which a cancer antigen-directed T cell receptor (TCR) may recognize a cardiac antigen that bears structural similarity at baseline or following posttranslational modification [5].

The TCR repertoire encompasses the cumulative TCR diversity exhibited by all T cells within an individual. The composition of an individual’s TCR repertoire is distinct, influenced by their genetic predisposition and environmental circumstances. This diversity plays a crucial role in combating intricate diseases and infections. In addition, TCR repertoire is not static, T cell clones expand when stimulated by antigen they recognizing, and contract after clearance of the antigen [6]. Hence, the investigation of the TCR repertoire holds considerable academic importance in elucidating the underlying mechanism of ICIs-associated myocarditis.

The Complementary Determining Region 3 (CDR3) is a critical region involved in antigen binding. The CDR3 domain is known to be a major contributor to sequence diversity and variability in TCRs. Specifically, the CDR3 region is located on the β chain and serves as the site for antigen recognition and binding [7]. TCR sequencing method mostly involves sequencing of CDR3 region on TCR β chain [8].

We hypothesized that TCR repertoire reconstituted exert a critical function in the pathogenesis and pathophysiology of ICIs-associated myocarditis. In this study, next generation sequencing (NGS) was used to monitor the expression pattern and clonality of CDR3 in ICIs-associated myocarditis patients. The identification of the TCR repertoire enhanced our comprehension of the underlying mechanisms and facilitates the identification of high-risk populations.

## Participants

In our hospital, a cohort of patients who experienced ICIs-associated myocarditis patients while undergoing treatment for malignant tumors with ICIs was collected between October 2022 and July 2023. According to the International Cardio-Oncology Society (IC-OS) 2021 consensus criteria [9], ICIs-associated myocarditis was divided into pathohistological diagnosis and clinical diagnosis, as shown in supplementary Table (1) Clinical diagnosis need to be met: A troponin elevation (new, or significant change from baseline) with 1 major criterion or a troponin elevation (new, or significant change from baseline) with 2 minor criteria after exclusion of acute coronary syndrome or acute infectious myocarditis based on clinical suspicion. The diagnostic basis of ICIs-associated myocarditis were presented in supplementary Table (2) Because we were unable to obtain pre-medication peripheral blood samples from patients with myocarditis, we recruited five patients who were treated with immune checkpoint inhibitors but did not develop myocarditis as the reference control group. Additionally, we included 30 healthy volunteers as the blank control group. The CDR3 repertoire data of the 30 healthy volunteers was obtained from Igenecode Biological Co., Ltd. (Beijing).

### Laboratory tests

Peripheral blood samples, ECG, and echocardiography were collected from patients diagnosed with myocarditis within 24 h of symptom onset. Two slightly symptomatic patients underwent testing the day after troponin T elevation was detected. Only three patients consented to undergo cardiac magnetic resonance imaging (MRI), while none agreed to undergo a cardiac biopsy.

### TCR repertoire library preparation and data processing

High quality gDNA were extracted from the fresh peripheral blood samples using HiPure Blood DNA Mini kit (MAGEN, Cat. no. D3111-03). Two steps of PCR were used to enrich the rearranged TCR gene fragments using 300–1,200 ng gDNA. The first step PCR is a multiplex PCR, and it goes 28 cycles, which includes forward primers located at the TRB/TRD/TRG/IGH/IGK/IGL variable genes and reverse primers located at the TRB/TRD/TRG/IGH/IGK/IGL joining genes. The second step PCR is a simplex PCR using a universal primer and goes 12 cycles, which brings in the whole adaptor sequence for Ilumina-Nova-seq platform to generate sequencing libraries. The sequencing libraries were sequenced on Ilumina-Nova-seq platform with a pair-end 150-bp reads. The raw sequencing reads were processed using IMonitor, and the CDR3s in a sample with the frequency of < 3 in 1 million sequences were filtered to remove the CDR3s containing sequencing errors.

The data obtained from sequencing is referred to as raw reads or raw data, which undergo quality control (QC) to assess their suitability for further analysis. As shown in supplementary Fig. 1, the filtered clean reads were subjected to comparison with the reference sequence. Subsequently, the matched reads were assembled to acquire specific functional regions. For instance, clone sequences exhibiting the desired base quality within the CDR3 region (clones) were employed as the core clonotype. Clones containing multiple bases with inadequate mass value were then compared and rectified by utilizing the core clone as a reference. Subsequently, the clones exhibiting a single base disparity underwent hierarchical clustering, wherein each branch was characterized by a sole base difference (mismatch). Clones with low cloning frequency were amalgamated into the preceding branch, while the principal head sequence was preserved. Subsequently, the acquired clone sequences were reevaluated against the V, D, J, and C reference sequences. The ultimate statistical file encompassed the clone sequence, amino acid residue sequence, clone number, clone frequency, V-J gene combination.

### Statistical analysis

The laboratory tests before and after the onset of immune checkpoint inhibitor-associated myocarditis were analyzed using a Paired *T*-test. The TCR diversity analysis and the examination of V and J gene segment usage patterns among the three groups were conducted using one-way analysis of variance (one-way ANOVA). All statistical analyses were performed using GraphPad Prism 9.5.1 (GraphPad Software, La Jolla, CA). Data were presented as the mean ± standard deviation. Significance was accepted at *P* < 0.05.

## Results

### Clinical characteristics of the patients

Peripheral blood TCR repertoire sequencing was conducted on 42 cases, including 7 patients diagnosed with ICIs-associated myocarditis, 5 patients demonstrated absence of myocarditis following a minimum of two cycles of ICIs, while the remaining 30 individuals without tumors served as healthy volunteers. The demographic characteristics and clinical features of the patients are presented in Table 1. Within group 1, comprising of six male and one female patients aged range 68 from 77 years, five patients received PD-1 inhibitors, while two were administered PD-L1 inhibitors. These individuals experienced immune checkpoint associated myocarditis within a range of 27 to 52 days post-administration, with an average onset time of 31 days. Based on the CTCAE 5.0 criteria, within group 1, two patients experienced grade 1 adverse events, two patients experienced grade 2 adverse events, and three patients experienced grade 3 or higher adverse events, one fatality due to ICIs-associated myocarditis.

As a reference control, the selection criteria for comorbidities in group 2 were made as comparable as feasible to those in group 1, as illustrated in Table 1. Among the five patients in group 2, consisting of one female and four males, who were administered immune-checkpoint inhibitors such as PD-1, PD-L1, and PD-1 combined with TIGIT blockade, no instances of myocarditis were observed.

### Changes of laboratory examination before medication and at onset of myocarditis

**Fig. 1:**
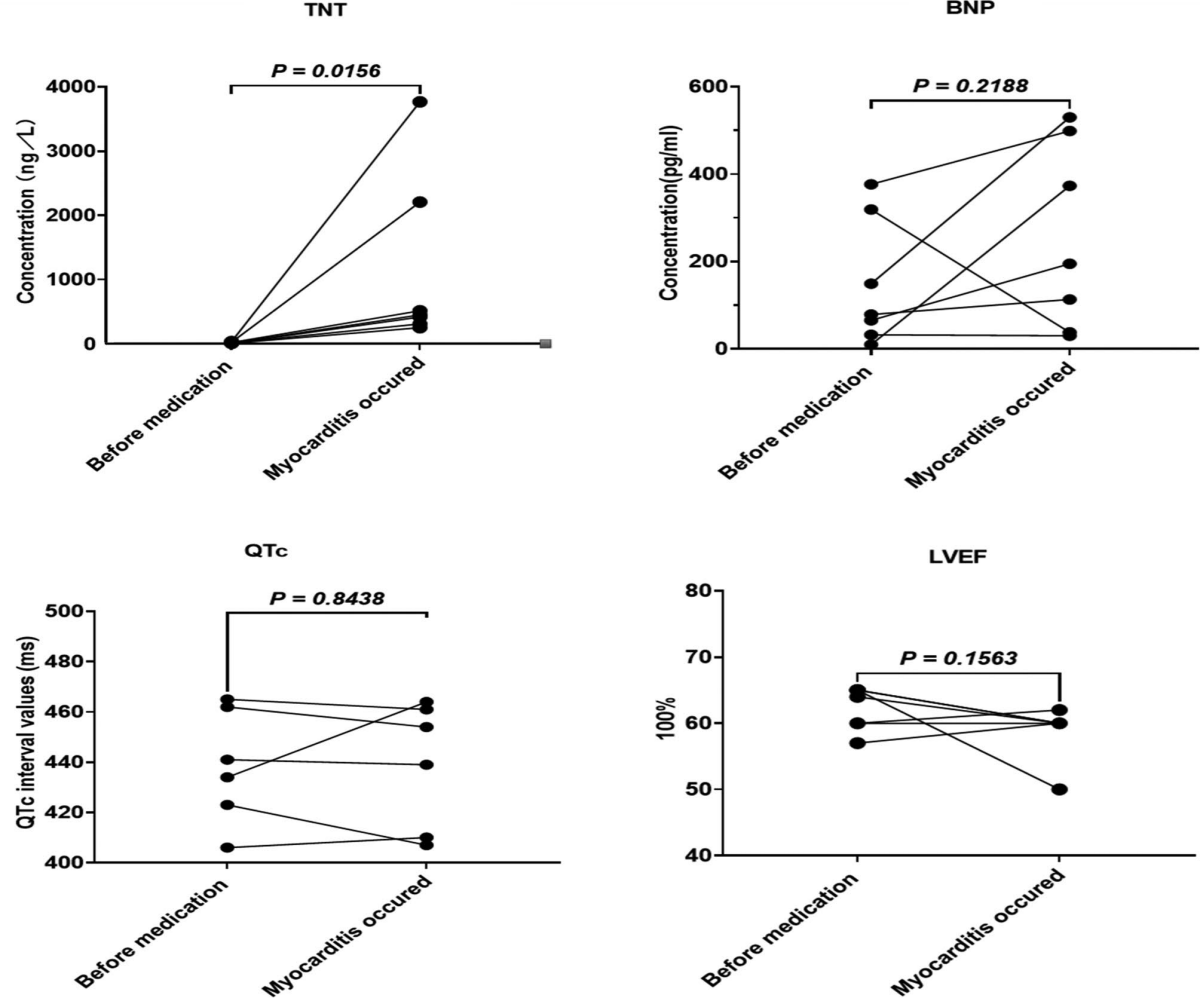
Changes of laboratory results before and after ICIs-associated myocarditis. The changes of troponin T, BNP, QTc interval and LVEF occurred before and after myocarditis

Troponin T (TNT), brain natriuretic peptide (BNP), ECG, and echocardiography were collected from patients both before medication and at the onset of myocarditis. The findings indicated a significant alteration in troponin T levels before and after myocarditis in the 7 patients (*P* = 0.0156). However, no significant changes were observed in BNP, QTc interval, and left ventricular ejection fraction (LVEF), as shown in Fig. 1.

**Table 1:** Patient characteristics

Group	Patients Number	Age	Gender	Main Diagnosis	Comorbidity	ICIs	Time from Initial Medication to Onset (days)	Symptom	ECG Changes	Adverse Effects Grade
1	1	73	Male	Colon cancer	None	PD-1	27	Fatigue	No change	G1
	2	68	Male	Esophageal cancer	Hypertension	PD-1	24	Fatigue	No change	G1
	3	69	Male	NSCLC	Hypertension, CHD, Heart Failure	PD-L1	52	Dyspnea, Palpitation	T wave depression	G3
	4	71	Male	SCLC	PAF, Hypertension, CHD	PD-L1	40	Dyspnea, Myasthenia gravis	T wave depression	G2
	5	70	Male	NSCLC	Hypertension, TDM2	PD-1	42	Dyspnea	Ventricular tachycardia, Preexcited ventricular responses	G5
	6	76	Male	EGJ cancer	Hypertension, Cerebral Infarction	PD-1	31	Dyspnea, Myasthenia gravis	Third-degree atrioventricular block, Ventricular escape beats	G4
	7	77	Female	Ureter cancer	Hypertension , CHD, TDM2	PD-1	28	Dyspnea, Myasthenia gravis	T wave depression	G2
2	8	49	Female	Gastric Cancer	None	PD-1	NA	NA	NA	NA
	9	56	Male	NSCLC	Emphysema	PD-L1	NA	NA	NA	NA
	10	69	Male	Laryngocarcinoma	PAF, ILD	PD-1+ TIGIT	NA	NA	NA	NA
	11	73	Male	Gastric cancer	Hypertension, CHD, Cerebral Infarction	PD-1	NA	NA	NA	NA
	12	67	Male	Gastric cancer	Hypertension	PD-1	NA	NA	NA	NA

Note: NSCLC: Non Small Cell Lung Cancer, SCLC: Small Cell Lung Cancer, EGJ: Esophagogastric Junction, CHD: Coronary Heart Disease, PAF: Paroxysmal Atrial Fibrillation, TDM2: Type 2 Diabetes Mellitus, ILD: Interstitial Lung Disease, NA: Not Applicable

### Diversity analysis of TCR CDR3 immune repertoire

**Fig. 2:**
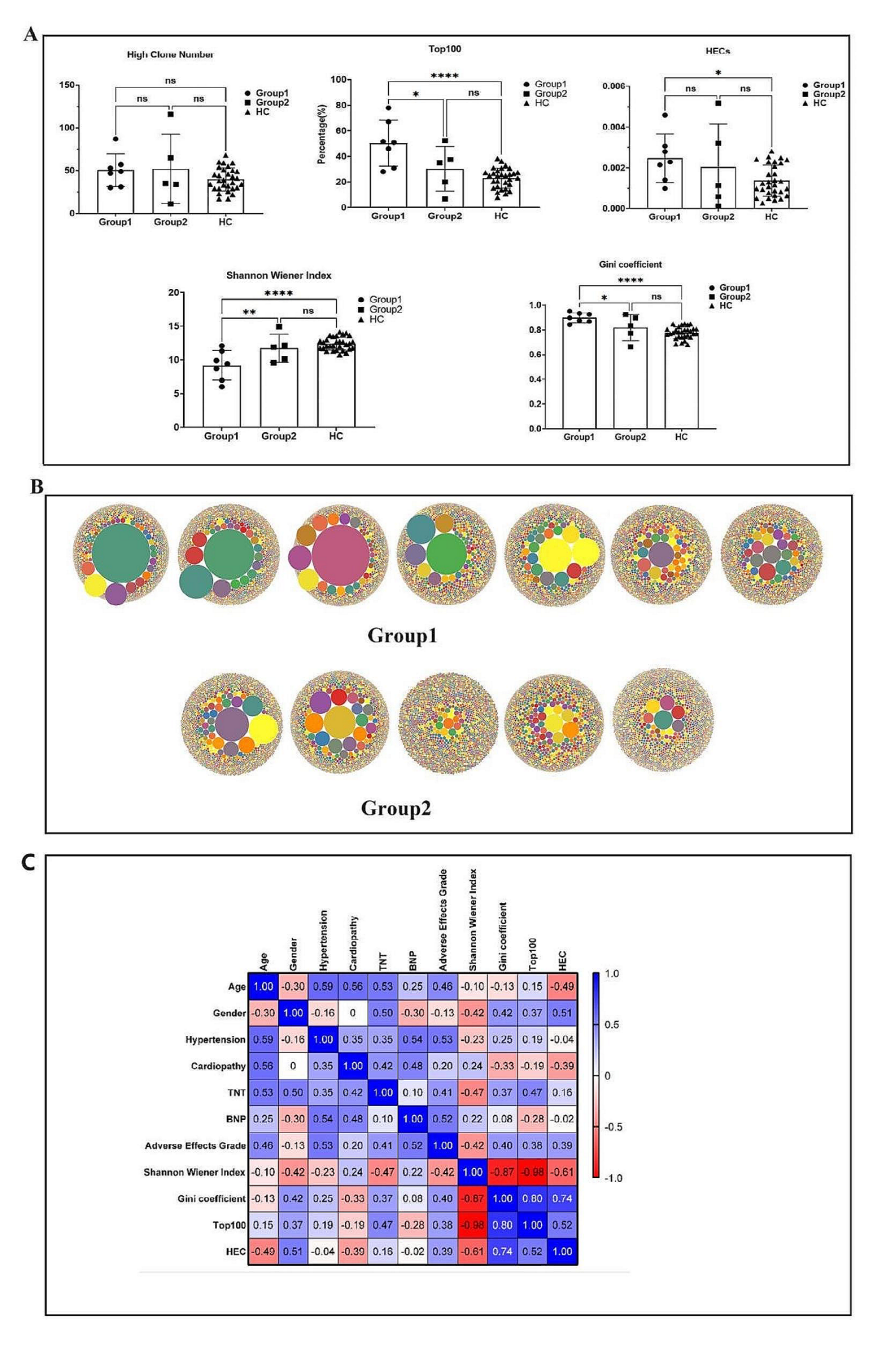
Diversity analysis of TCR CDR3 immune repertoire. **(A)** High clone number, HECs, TOP 100 clones, Shannon Wiener index and Gini coefficient in different groups. **(B)** Diversity results diagram. Each circle in the figure represents a unique clone, and the size of the circle was proportional to the frequency of the clone. **(C)** Correlation of CDR3 diversity vs. other clinical markers. Red: positive correlation; blue: negative correlation. Gender, history of hypertension and history of cardiopathy were categorical variable (1 for male and 2 for female). There was a history of hypertension (1) and no history of hypertension (0). Having a history of cardiopathy was 1, and vice versa was 0. Group 1: Patients with ICIs-associated myocarditis, Group 2: Patients without ICIs-associated myocarditis; HC group: Healthy individuals control. *P < 0.05, **P < 0.01, ***P < 0.001, ****P < 0.0001

In order to assess the clonal diversity of CDR3, various indicators such as Unique clone number, highly Expanded Clones (HECs), Shannon Wiener Index, and Gini coefficient were employed [10]. The number of HECs was determined by dividing the total number of clones by the high number of unique clones. As shown in Fig. 2a, there was no significant difference in the number of high clones number among the three groups. Top 100 clone refer to the cumulative frequencies of the top 100 clones [11], the top 100 clones was a statistically significant difference observed among the three groups. Specifically, the number of TOP100 clones in group 1 was significantly higher compared to group 2 (*P* = 0.011) and the healthy individuals control (HC) group (*P* < 0.0001). Additionally, in group 1, the HECs were significantly higher than the HC group (*P* = 0.046), while no statistical difference was observed between group 1 and group 2 (*P* = 0.77). The Shannon Wiener Index was employed to quantify clonal diversity, with higher values indicating greater diversity. Group 1 exhibited significantly lower diversity than both group 2 (*P* = 0.0075) and the HC group (*P* < 0.0001). The Gini coefficient, utilized to measure clone equilibrium, ranges from 0 (perfectly average data distribution) to 1 (completely uneven distribution). Group 1 demonstrated significantly higher Gini coefficients compared to both group 2 and the HC group, with *P* values of 0.043 and < 0.0001, respectively.

The diversity results of each sample were depicted in Fig. 2b through a schematic diagram. In this visual representation, individual clones were denoted by circles, with the size of each circle corresponding to the clone’s frequency. A higher diversity of an individual clone was indicated by a more evenly distributed frequency. Additionally, the schematic diagram revealed that the ICIs-associated myocarditis group exhibited a greater number of high clones, with a more uneven distribution.

The relationship between CDR3 diversity and clinical parameters of patients was illustrated in Fig. 2c. CDR3 diversity did not exhibit a strong correlation with variables such as age, gender, history of hypertension and cardiopathy, levels of TNT and BNP, and severity of adverse reactions at the onset of symptoms.

### Distribution characteristics of CDR3 nucleotide length

**Fig. 3:**
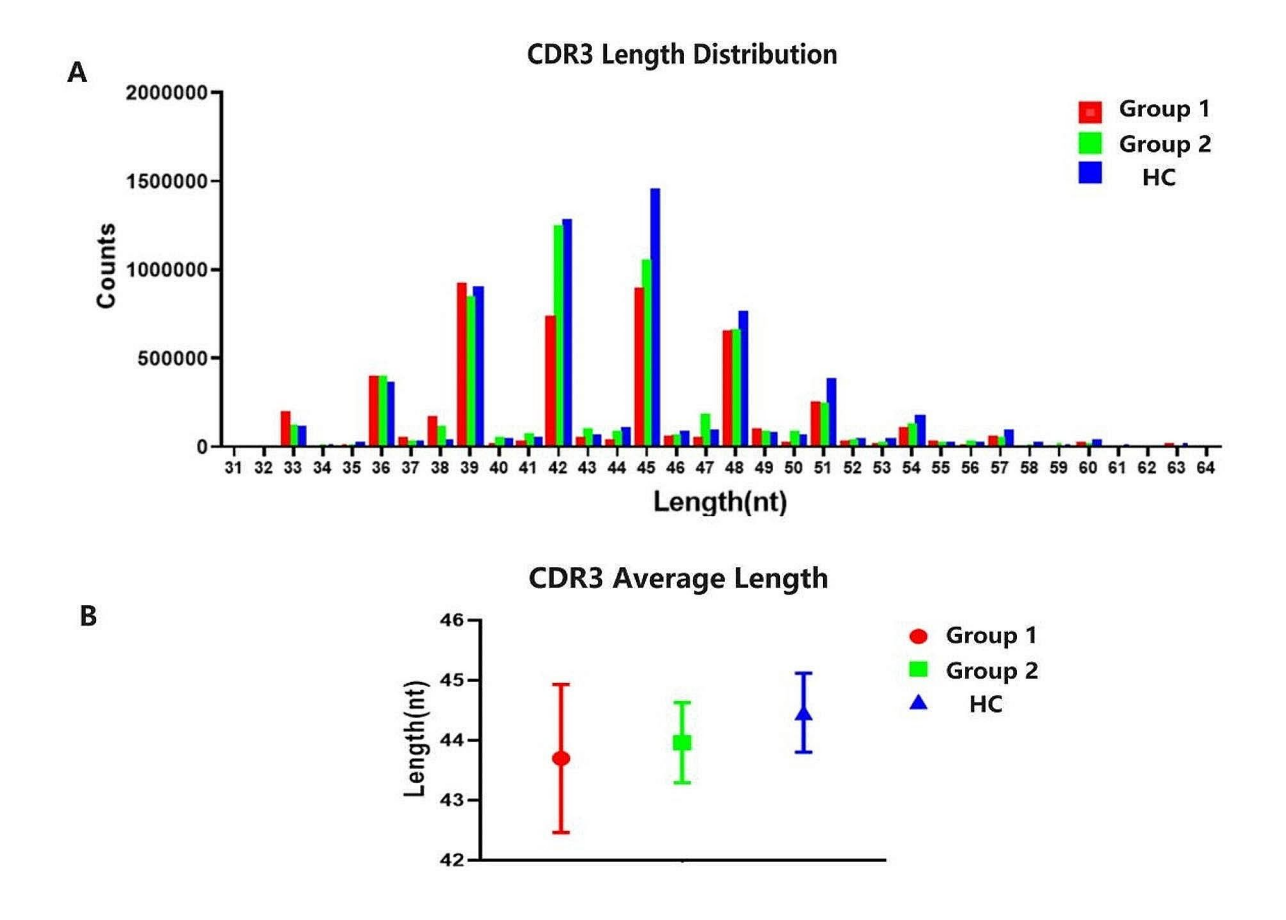
Distribution characteristics of CDR3 length. **(A)** CDR3 length distribution among the three different groups. **(B)** The average length of CDR3 among the three different groups. There was no statistical difference among the three groups. Group 1: Patients with ICIs-associated myocarditis, Group 2: Patients without ICIs-associated myocarditis; HC group: Healthy individuals control

The CDR3 region typically consisted of a range of 10 to 20 amino acids, the distribution of nucleotides ranged from about 21 to 81 nucleotide (nt), with 45 nt was peak [12], and its length distribution served as a crucial indicator for identifying TCR polymorphism and reflecting the distribution of T cell clones. The length distribution of CDR3 can provide insights into the overall state of the immune system, while average length of CDR3 sequence also affected the immune diversities [13]. As shown in Fig. 3a&b, in healthy individuals (group HC), the distribution of CDR3 exhibited a normal distribution with a mean length of 44.46 ± 0.66 nucleotides and a peak at 45 nucleotides. Conversely, individuals with ICIs-associated myocarditis (group 1) displayed a bimodal distribution with peaks at 39 and 45 nucleotides. The mean length in this group was 43.69 ± 1.24 nucleotides. Tumor patients without myocarditis (group 2) also exhibited an abnormal distribution, with a peak at 42 nucleotides and a mean length of 43.96 ± 0.67 nucleotides.

### The usage patterns of V and J gene segments

**Fig. 4:**
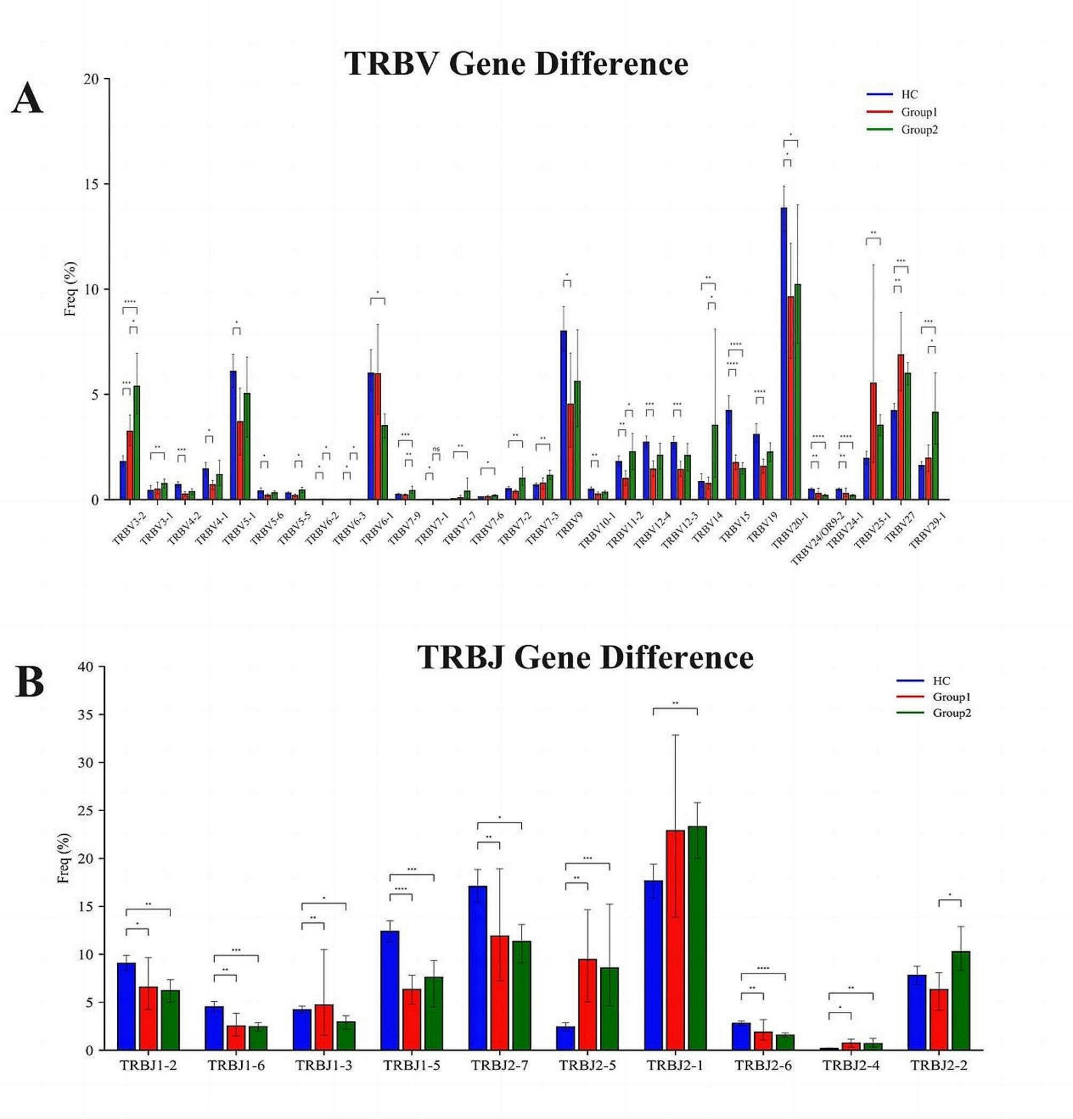
The usage patterns of V **(A)** and J gene **(B)** segments. Group 1: Patients with ICIs-associated myocarditis, Group 2: Patients without ICIs-associated myocarditis; HC group: Healthy individuals control. *P < 0.05,**P < 0.01,***P < 0.001

The diversity of V and J genes contributed to the overall diversity of the immune repertoire, with individuals exhibiting varying frequencies of V and J gene usage. Investigating these frequency differences was crucial for elucidating variations within the sampled immune repertoire. Consequently, statistical analyses were performed to determine the frequencies of V and J genes in each sample. As shown in Fig. 4a&b, compared with group 2, it was that decreased clonal expansion frequencies of the TRBV3-2,TRBV5-5,TRBV7-9, TRBV11-2, TRBV14, TRBV29-1 and TRBJ2-2 were determined in ICIs-associated mycarditis patients.

**Fig. 5:**
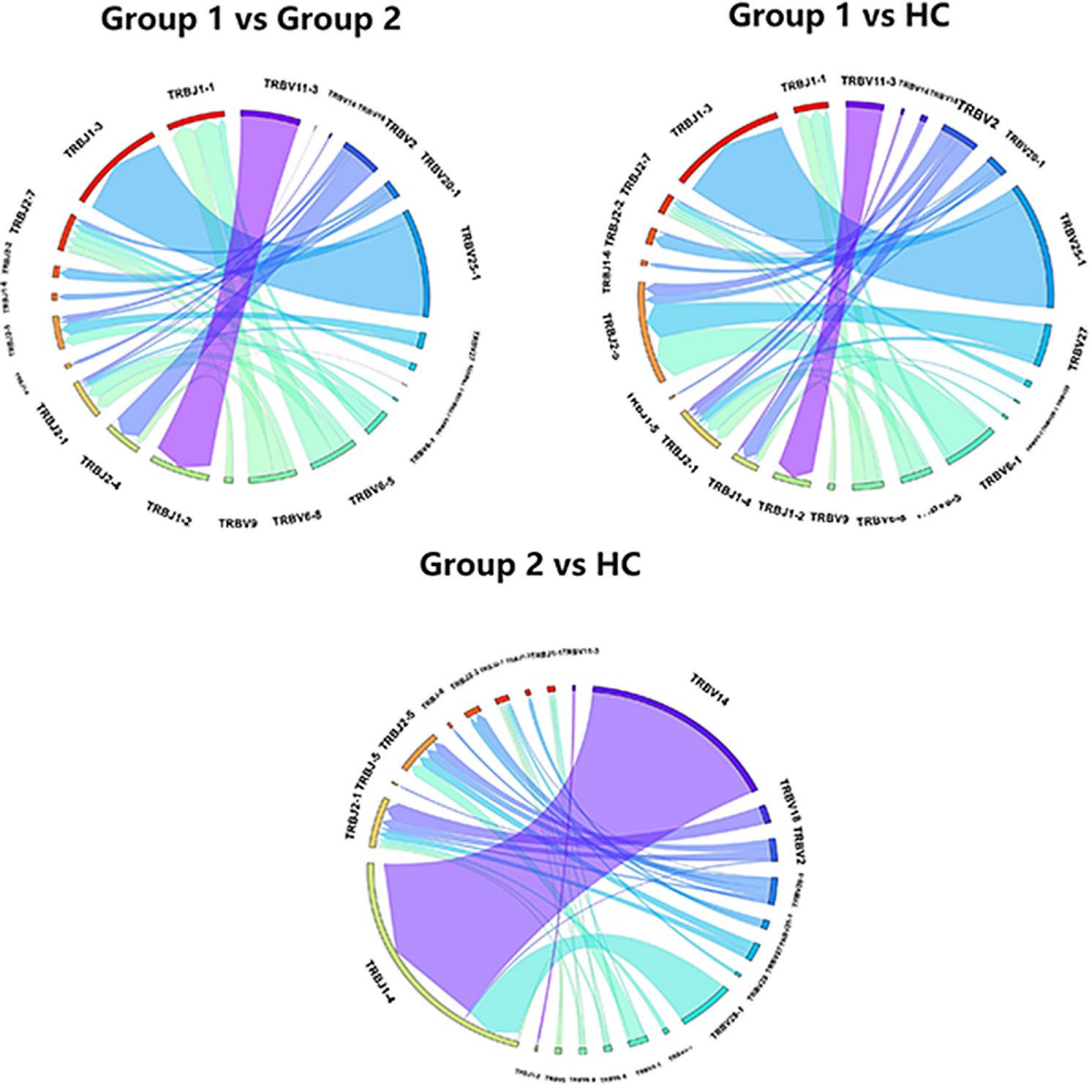
Differential V-J combination distribution circular plots. Each color block represents a gene, and the wider the color band, the higher the frequency. The lines between the blocks represented a combination of V-J genes. Group 1: Patients with ICIs-associated myocarditis, Group 2: Patients without ICIs-associated myocarditis; HC group: Healthy individuals control

The diversity of V-J gene combinations served as an indicator that highlighted the variations observed among the samples. We conducted a comprehensive analysis by quantifying the occurrence of cloned V-J gene combinations in each sample and determining the corresponding frequencies for each combination. Through rigorous statistical analysis, we observed that each group expressed no fewer than three distinct V-J gene combinations, and we identified combinations that exhibited significant differences between the groups (*P* < 0.05). The circular plots depicting the various combinations within each sample was presented in Fig. 5. Each gene was represented by a distinct color block, with the width of the block indicating its frequency. The lines connecting the blocks represented the combinations of V-J genes. In comparison to tumor patients without myocarditis, myocarditis patients displayed 24 V-J combinations that demonstrated significantly usage. Among these combinations, the most frequent pairing was TRBV25-1 with TRBJ1-3, followed by TRBV11-3 with TRBJ1-2. In contrast, when compared to healthy individuals, the V-J gene combination with the highest frequencies remained TRBV25-1 paired with TRBJ1-3, while the subsequent highest combination was TRBV6-1 paired with TRBJ2-5.

## Discussion

Previous studies had established that the histopathological features of ICIs-associated myocarditis involved the infiltration of T lymphocytes into the myocardium [14]. It had been also reported that irAEs were linked to TCR diversification subsequent to treatment [15, 16]. As far as we known, our study was first time found that the TCR immune repertoire reconstituted at the onset of ICI-associated myocarditis, identifying and tracking TCR immune repertoires provided a novel method to understand the association between T lymphocytes and ICIs-associated myocarditis.

Both CDR1 and CDR2 regions are encoded by germline V genes, whereas the CDR3 region was formed by the somatic rearrangement of V, (D) and J fragments, which embody TCR diversity [17]. CDR3 Sequences from T-Cell Receptor β could be stimulated by specific antigens to produce rapid reconstitution as an adaptive immune response when certain diseases occurred [18–20]. This study aimed to investigate the characteristics of the TCR CDR3 immune repertoire in peripheral blood in individuals with ICIs-associated myocarditis. Due to the unavailability of pre-medication peripheral blood samples from patients, the patients consisting of individuals without myocarditis who received ICIs, and who were matched in terms of age and underlying comorbidities were as control group.

Our findings indicated that the Shannon Wiener Index in ICIs-associated myocarditis patients was significantly lower compared to both the group without myocarditis and healthy individuals control. Additionally, the Gini coefficient and Top100 clone in the ICIs-associated myocarditis group were significantly higher than those observed in the group without myocarditis and healthy individuals control. These results collectively suggest that the diversity of immune repertoire in the ICIs-associated myocarditis group was reduced, while its distribution was more uneven. Accumulating evidence demonstrate that TCR repertoires would be rapidly reconstituted in response to endogenous and exogenous stimuli for immunosurveillance and immunoregulation [21–23]. One study showed that decreased TCR diversity reflected reduced peripheral immune surveillance [24]. We also observed a significant increase in HECs in patients with ICIs-associated myocarditis. The increase in HECs was also observed in other diseases, such as systemic lupus erythematosus and rheumatoid arthritis, indicating that the increase of HECs might be a common phenomenon in autoimmune diseases [25].

Considering the parameters aforementioned, we proposed the hypotheses that the induction of antigen-stimulated T cell differentiation by ICIs lead to a reduction in diversity, impairment of immune surveillance capability, and subsequent development of chronic immune imbalance and aberrant autoimmune responses.

The sequence length variation and distribution characteristics of the CDR3 region of TCR could provide a line for studying the structure-function relationship of different TCR in recognizing specific antigens [26]. The length of CDR3 varies from 21 to 81 nt with a peak at 45 nt [12]. It has been shown that typical histograms that were derived from mature peripheral T lymphocyte pools displayed a normal distribution with 3-base spacing. If a histogram is biased by an unexpectedly high frequency at a specific length, it indicated that the studied population contains an expanded T cell clone whose CDR3 has the corresponding length [27]. The CDR3 distribution in the tumor group without myocarditis maintained a normal pattern, despite a reduction of a few nucleotides at peak (45nt vs. 42nt). Conversely, the length distribution of CDR3 in the ICIs-associated myocarditis group deviated from normality and exhibited greater disorder. This suggested that T cells expressing diverse amino acid sequences in the β-chain CDR3 region might contribute to the immune response against myocarditis antigens.

The diversity of V and J genes contributed to the diversity observed in the immune repertoire. Variations in the frequencies of V and J genes among individuals hold significant implications for elucidating the disparities within the immune repertoire of different samples. In this study, it was that the decreased clonal expansion frequencies of the TRBV3-2,TRBV5-5,TRBV7-9, TRBV11-2, TRBV14, TRBV29-1 and TRBJ2-2 were determined in ICIs-associated mycarditis patients. This disparity had also been documented in alternative forms of myocarditis. The TRBV exhibited significant variations across different viral myocarditis cases, specifically, *Parvovirus B19* infection was associated with increased expression of TRBV 11 and 24, while *human herpes virus type 6* infection was linked to elevated levels of TRBV4, 10, and 28. Additionally, *Coxsackie* virus infection was found to be associated with increased expression of TRBV14 [28]. Notably, patients with acute myocardial infarction displayed high clonal expansion frequencies of TRBV10-3, whereas TRBV11-2 exhibited low clonal expansion frequencies along with TRBV9, TRBV3-1, TRBV6-7, and TRBJ5-1 [18]. In the context of ICIs-associated myocarditis, we had identified a novel disparity in the utilization of the V and J genes, marking the first instance of such observation. The clonal expansion of V-J genes combination had been observed to induce immune activation in T cells. This study also observed gene recombination of V and J genes in the CDR3 region among patients diagnosed with myocarditis, leading to alteration of diversity.

Nonetheless, this present study had some limitations. The number of cases collected in a single center was limited, leading to a very small sample size. Additionally, peripheral blood was not collected prior to myocarditis, thereby preventing observation of the dynamic changes in individual TCR repertoire. We plan to establish biobanking to address this issue.

## Conclusions

Our study provides novel insights into the characteristics of the TCR immune repertoire following the onset of ICIs-associated myocarditis. By closely monitoring the reconstruction process of the TCR immune repertoire, we observed a reduction in the diversity of the CDR3 region, the absence of the previously observed normal distribution of amino acids, and the identification of specific alterations in the utilization and recombination of V-J genes. These significant findings strongly support the notion that the modification of TCR immune repertoire plays a critical role in antigen recognition within the adaptive immune system of myocarditis patients.

### Electronic supplementary material

Below is the link to the electronic supplementary material.

Supplementary Material 1

Supplementary Material 2

Supplementary Material 3

## Data Availability

No datasets were generated or analysed during the current study.
