# Supplementary figures and images for: Reconstitution of peripheral blood T cell receptor β immune repertoire in immune checkpoint inhibitors associated myocarditis
Source: Cardiooncology. 2024 Jun 11;10:35. doi: 10.1186/s40959-024-00230-4 (PMC11165862; doi:10.1186/s40959-024-00230-4)

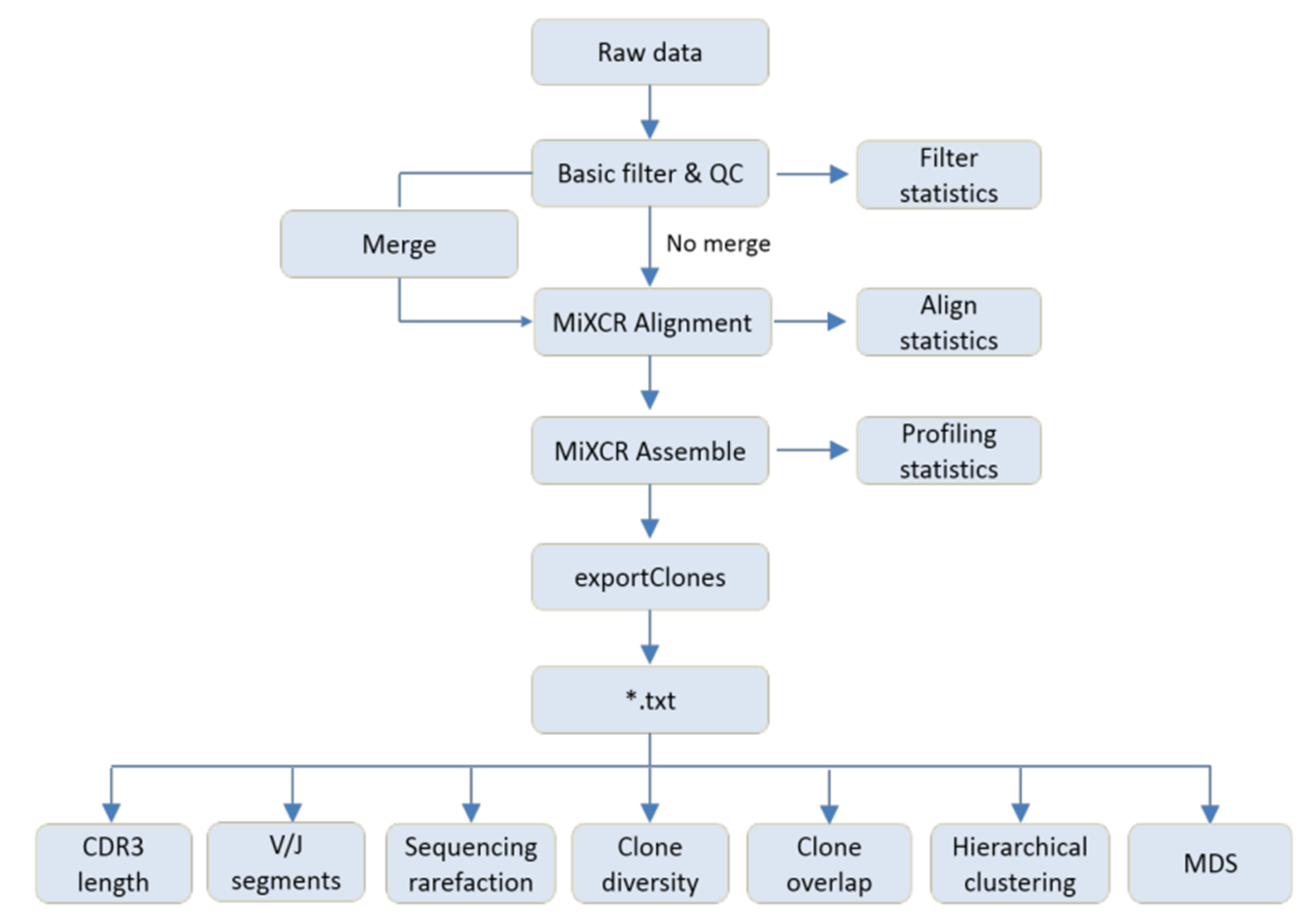

Supplement: Supplementary file 1 — Supplementary Material 1 [file 40959_2024_230_MOESM1_ESM.tif]
