# Supplementary material for: Reconstitution of peripheral blood T cell receptor β immune repertoire in immune checkpoint inhibitors associated myocarditis
Source: Cardiooncology. 2024 Jun 11;10:35. doi: 10.1186/s40959-024-00230-4 (PMC11165862; doi:10.1186/s40959-024-00230-4)
Supplement: Supplementary file 2 — Supplementary Material 2 [file 40959_2024_230_MOESM2_ESM.docx]

**Supplementary Table 1**

**Deﬁnitions for Immune Checkpoint Inhibitor Associated Myocarditis**

| **IC-OS 2021 Consensus** |
| --- |
| **Either pathohistological diagnosis:** Multifocal inﬂammatory cell inﬁltrates with overt cardiomyocyte loss by light microscopy of cardiac tissue samples.  **Or clinical diagnosis**: A troponin elevation (new, or signiﬁcant change from baseline) with 1 major criterion or a troponin elevation (new, or signiﬁcant change from baseline) with 2 minor criteria after exclusion of acute coronary syndrome or acute infectious myocarditis based on clinical suspicion.  **Major Criterion:** CMR diagnostic for acute myocarditis (modiﬁed Lake Louise criteria)  **Minor Criteria:**   - Clinical syndrome (including any one of the following: fatigue, muscle weakness, myalgias, chest pain, diplopia, ptosis, shortness of breath, orthopnea, lower extremity edema, palpitations, lightheadedness/dizziness, syncope, cardiogenic shock) - Ventricular arrhythmia and/or new conduction system disease. - Decline in cardiac (systolic) function, with or without regional WMA in a non-Takotsubo pattern - Other immune-related adverse events, particularly myositis, myopathy, myasthenia gravis - Suggestive CMR (meeting some but not all of the modiﬁed Lake Louise citeria) |

Note:CMR:cardiac magnetic resonance; WMA: wall motion abnormalities
