# Supplementary material for: Reconstitution of peripheral blood T cell receptor β immune repertoire in immune checkpoint inhibitors associated myocarditis
Source: Cardiooncology. 2024 Jun 11;10:35. doi: 10.1186/s40959-024-00230-4 (PMC11165862; doi:10.1186/s40959-024-00230-4)
Supplement: Supplementary file 3 — Supplementary Material 3 [file 40959_2024_230_MOESM3_ESM.docx]

**Supplementary Table 2 Diagnostic Basis of ICIs-associated Myocarditis**

| **Patients Number** | **Myocardial Biopsy** | **CMR** | **Syndrome** | **Angiography/**  **Coronary CTA** | **ECG** | **Troponin T** | **Other Immune-related Adverse Events** | **Sensitivity to Glucocorticoid Treatment** | **Co-infection** |
| --- | --- | --- | --- | --- | --- | --- | --- | --- | --- |
| 1 | Not done | Not done | Fatigue | CTA:  Mild coronary stenosis | Non-ACS ECG | Significantly increase | None | Sensitive | No |
| 2 | Not done | Left ventricular wall myocardial injury/early myocardial fibrosis | Fatigue | Not done | Non-ACS ECG | Significantly increase | Hypocorticoidism | Sensitive | No |
| 3 | Not done | The myocardium of the left ventricular septal wall exhibits edema, injury, and fibrosis | Dyspnea,  Palpitation | Not done | Non-ACS ECG | Significantly increase | Hypocorticoidism  Hypothyroidism | Sensitive | No |
| 4 | Not done | Not done | Dyspnea | Not done | Non-ACS ECG | Significantly increase | Myasthenia gravis | Sensitive | No |
| 5 | Not done | Left ventricular basal septum edema and thickening | Dyspnea | Not done | Ventricular tachycardia,  Preexcited ventricular responses | Significantly increase | None | Sensitive | No |
| 6 | Not done | Not done | Dyspnea | Angiography:  TIMI grade 3 flow | Third-degree  atrioventricular block,  Ventricular escape beats | Significantly increase | Myasthenia gravis | Sensitive | No |
| 7 | Not done | Not done | Dyspnea | Not done | Non-ACS ECG | Significantly increase | Myasthenia gravis | Sensitive | No |

Note: CMR: cardiac magnetic resonance; ECG: electrocardiogram; ACS: acute coronary syndrome; TIMI: thrombolysis in myocardial infarction.

# **Supplementary Table 2 continued**

| **Patients Number** | **The diagnosis based on IC-OS 2021 Consensus** |
| --- | --- |
| 1 | Troponin elevation+2 minor criteria (fatigue, LVEF decreased) ,  exclusion of ACS or acute infectious myocarditis based on clinical suspicion |
| 2 | Troponin elevation+4 minor criteria(fatigue, elevated BNP, suggestive CMR, hypocorticoidism) ,  exclusion of ACS or acute infectious myocarditis based on clinical suspicion |
| 3 | Troponin elevation+4 minor criteria (dyspnea and palpitation, elevated BNP and LVEF decreased, suggestive CMR, hypocorticoidism and hypothyroidism) ,  exclusion of ACS or acute infectious myocarditis based on clinical suspicion |
| 4 | Troponin elevation+3 minor criteria(dyspnea, elevated BNP, myasthenia gravis) ,  exclusion of ACS or acute infectious myocarditis based on clinical suspicion |
| 5 | Troponin elevation+4 minor criteria (dyspnea, ventricular tachycardia and  preexcited ventricular responses, LVEF decreased, suggestive CMR),  exclusion of ACS or acute infectious myocarditis based on clinical suspicion |
| 6 | Troponin elevation+3 minor criteria (dyspnea, third-degree atrioventricular block and ventricular escape beats, myasthenia gravis),  exclusion of ACS or acute infectious myocarditis based on clinical suspicion |
| 7 | Troponin elevation+2 minor criteria (dyspnea, myasthenia gravis),  exclusion of ACS or acute infectious myocarditis based on clinical suspicion |

Note:LVEF: Left ventricular ejection fraction; BNP:Brain natriuretic peptide.
